# Comprehensive analysis of mRNA‐level and miRNA‐level subpathway activities for identifying robust ovarian cancer prognostic signatures

**DOI:** 10.1111/jcmm.14968

**Published:** 2020-01-19

**Authors:** Songyu Tian, Wanqi Mi, Mingyue Zhang, Linan Xing, Chunlong Zhang

**Affiliations:** ^1^ Department of Gynecological Oncology Harbin Medical University Cancer Hospital Harbin China; ^2^ College of Bioinformatics Science and Technology Harbin Medical University Harbin China; ^3^ Department of Anesthesiology Harbin Medical University Cancer Hospital Harbin China

**Keywords:** integrated analysis, ovarian cancer, prognostic signature, subpathway activity

## Abstract

Ovarian cancer (OvCa) causes the highest mortality among all gynaecologic cancers. A large number of mRNA‐ or miRNA‐based signatures were identified for OvCa patient prognosis. However, the comprehensive analysis of function‐level prognostic signatures is currently not considered in OvCa. In the present study, we respectively inferred subpathway activities from mRNA and miRNA levels based on high‐throughput expression profiles and reconstructed subpathways. Firstly, the activities of two tumour pathways were calculated and the difference between normal and tumour samples were analysed using multiple tumour types. Then, we calculated subpathway activities for OvCa based on the expression profiles from both mRNA and miRNA levels. Furthermore, based on these subpathway activity matrices, we performed bootstrap analysis to obtain sub‐training sets and utilized univariate method to identify robust OvCa prognostic subpathways. A comprehensive comparison of subpathway results between these two levels was performed. As a result, we observed subpathway mutual exclusion trend between the levels of mRNA and miRNA, which indicated the necessary of combining mRNA‐miRNA levels. Finally, by using ICGC data as testing sets, we utilized two strategies to verify survival predictive power of the mRNA‐miRNA combined subpathway signatures and performed comparisons with results from individual levels. It was confirmed that our framework displayed application to identify robust and efficient prognostic signatures for OvCa, and the combined signatures indeed exhibited advantages over individual ones. In the study, we took a step forward in relevant novel integrated functional signatures for OvCa prognosis.

## 1 INTRODUCTION

Ovarian cancer (OvCa) is a widespread cancer that causes the highest mortality among all the gynaecologic cancers. And epithelial OvCa is the most common type accounting for about 90% of all cases.^1^ OvCa patients generally do not have symptoms or mild symptoms in their early stages. However, the patients in advanced stages will suffer from pelvic mass, abdominal distension, ascites and so on. Although advanced‐stage patients have initial responses to treatment, most of them will relapse, become resistant and even die. According to the International Federation of Gynecology and Obstetrics (FIGO) staging system and BRCA1/2 mutation status, clinical treatments for OvCa patients usually consist of surgery, chemotherapy and targeted therapy. However, current pre‐treatment evaluation methods are not adequate because of OvCa molecular heterogeneity. For the patients who belong to the same FIGO stage and BRCA1/2 status, extremely different clinical outcomes are often observed.^2^, ^3^ Therefore, gynaecologists need more specific and robust biological markers for prognosis analysis of OvCa patients.

MicroRNAs (miRNAs) are the most common non‐coding RNAs. Through binding to 3'‐untranslated regions of messenger RNAs (mRNAs) or other RNAs, miRNAs display crucial regulatory roles at the post‐transcriptional level.^4^ MiRNA‐related pathways play an important role in reprogramming mRNA expression in OvCa.^5^ Givel et al verified that the regulatory function of miR‐200 on CXCL12β could affect immuno‐suppression and fibroblast heterogeneity in OvCa.^6^ Bagnoli et al identified a miRNA‐based signature (MiROvaR) to successfully predict early relapse and progression of epithelial OvCa.^7^ Wu et al confirmed that the miR‐192‐EGR1‐HOXB9 regulatory axis was involved in the angiogenesis in OvCa.^8^ Furthermore, Au Yeung et al proved exosomal transfer of stroma‐derived miR‐21 could confer paclitaxel resistance of OvCa cells.^9^

To further deeply explore functional effects of miRNAs on malignant tumour development and progression, researchers have performed different kinds of integrated analyses at the miRNA and mRNA levels. In 2014, Calura et al developed an approach to wire miRNAs into pathways, dissecting the complex tumour regulatory networks through analysing high‐throughput miRNA and mRNA expression profiles.^10^ Roy et al performed an integrated analysis based on miRNA and mRNA expression levels in mouse and human hepatocellular carcinoma tissues. Through a series of experiments, these researchers confirmed that miR‐193a‐5p regulated the expression levels of NUSAP1 and further suppress hepatocarcinogenesis.^11^ Frampton et al combined miRNA and mRNA expression profiles of pancreatic ductal adenocarcinoma and normal samples to construct a miRNA‐mRNA regulatory network and identify some key miRNAs involved in pancreatic ductal adenocarcinoma.^12^ Tasena et al established a complex miRNA‐mRNA network for chronic mucus hypersecretion and identified several pivotal miRNAs and their potential target mRNAs as disease bio‐markers.^13^

In 2017, our group performed an integrated analysis of high‐throughput miRNAs’ and mRNAs’ expression to discern core OvCa prognostic subpathways using The Cancer Genome Atlas (TCGA) data set.^14^ Based on these prognostic subpathways, we further utilized random walk algorithm to assign a risking score to each miRNA and mRNA component, and final subpathway signatures were identified by ranking the overall score of both miRNA and mRNA components involved in this subpathway. Finally, we verified the predictive power of subpathway signatures for OvCa prognosis by using independent data sets from Gene Expression Omnibus (GEO) and International Cancer Genome Consortium (ICGC) databases. In this paper, we considered two different expression data sets and subpathway concept to identify functional signatures, and the identified subpathways which contained less gene components displayed more detailed functional description. Although this research displayed good performance, two issues are needed to be resolved for OvCa analysis. The first matter is that the training samples are randomly selected only once from TCGA, which is not beneficial to the reliability of signatures. The robustness of the signature should further be validated. Secondly, we treated the miRNA molecule as an independent component of the subpathway and did not consider its negative regulation on mRNA functions.

As is known, mRNA overexpression results in high activity level of the pathway in which these mRNAs are located. On the contrary, miRNAs’ overexpression results in the suppression of expression levels of corresponding target mRNAs, which lead to lower activity levels of corresponding pathway in which the target mRNAs are located. Therefore, systematic analyses and comparisons of subpathway functional conditions between mRNA level and miRNA level are urgently needed for OvCa prognosis. To improve these issues and increase reliability of prognostic signatures, we designed this project. We first utilized mRNA expression levels to calculate subpathway activity at the mRNA level (mRNA‐level subpathway activity) using the FAIME strategy^15^ in which mRNA expression rank in descending order. Owing to negative regulation of miRNAs, we used the FAIME method to calculate activity for the same subpathway from miRNA level (miRNA‐level subpathway activity) by considering a different direction, in which miRNA expression rank in ascending order. So, for mRNA and miRNA level, the subpathway activity matrix was respectively formed with subpathways as rows and tumour samples as columns. Secondly, we made disturbances of TCGA OvCa samples to obtain 1000 sub‐training sets, and further identified robust subpathways with high counts in all sub‐training sets from both mRNA level and miRNA level. On these grounds, we classified all prognostic subpathways into risk subpathways and protective subpathways. A kind of mutually exclusive trend of subpathway results was observed between mRNA level and miRNA level. Finally, using ICGC data as an independent validation set, we found that the combination of robust subpathway signatures from mRNA level and miRNA level displayed stronger predictive effect than mRNA‐level and miRNA‐level signatures, respectively.

## 2 MATERIAL AND METHOD

### 2.1 Training data set from TCGA

We obtained OvCa data set from TCGA as a training set which included molecular (miRNAs and mRNAs) expression profiles and patient clinical information. With regard to the molecular expression data, the miRNA expression was generated by BCGSC miRNA profiling, and mRNA expression was generated by HTseq‐FPKM. For reduplicative samples, we calculated average mRNA and miRNA expression values as final values. Furthermore, samples corresponding to patients with survival time less than 30 days were eliminated, because these patients may have died due to other reasons.^16^ The molecular expression data and corresponding survival events of a total of 370 OvCa patients were used in the entire training set. In addition to that, in order to identify the most robust prognostic signatures, we made 1000 disturbances to 370 OvCa samples and constructed 1000 sub‐training sets, which had the same ratio of sample survival distribution as the whole training set taking the median survival time as the cut‐off. For each sub‐training set, we utilized FAIME and modified FAIME methods to respectively construct mRNA‐level and miRNA‐level subpathway activity matrix, in which subpathway as rows and OvCa samples as columns.

### 2.2 Validation data set from ICGC

We obtained another available data set (OV‐AU) from ICGC database to test the prognostic performance of only miRNA‐level subpathways, only mRNA‐level subpathways as well as merged subpathways. From this independent data set, we also obtained miRNA, mRNA expression profiles and available clinical information of 93 OvCa samples. Performing the similar procedure with training set, we also formed an mRNA‐level and miRNA‐level subpathway matrix based on the sample‐matched mRNA and miRNA expression profiles. In the validation part, we adopt two strategies (rank‐based and threshold‐based) to verify prognostic reliability of the results obtained from the training set. We obtained the count value from training set from the 1000 random analysis, and a number from 1 to 1000 was assigned to each signature. Then, all the signatures were ranked in a descending order. The first one is to set a number K from 2 to 8 to define signatures that contained top K risk and protective subpathways. Another one is to set three different threshold numbers (450, 500 and 550) to define signatures. We tested the efficacy of all the selected subpathway signatures using the validation data set.

### 2.3 Other data sets from TCGA

To test the reliability of FAIME method for OvCa prognosis analysis, we tried to compare the difference of subpathway activity between normal and tumour samples. Due to the absence of normal sample of OvCa in TCGA database, we further obtained high‐throughput mRNA and miRNA expression profiles of seven other tumour types from TCGA, including bladder urothelial carcinoma (BLCA), breast invasive carcinoma (BRCA), colon adenocarcinoma (COAD), lung adenocarcinoma (LUAD), lung squamous cell carcinoma (LUSC), prostate adenocarcinoma (PRAD) and thyroid cancer (THCA). The mRNA and miRNA expression level from both tumour and normal samples were obtained and utilized in the method comparison. The subpathway activity was calculated for both tumour and normal samples, and the comparison was performed.

### 2.4 Subpathway data

We directly obtained subpathway data from R package *SubpathwayMiner*, in which researchers located subpathway regions from total pathways by using the distance similarity method.^17^ We also embedded miRNA molecules into the subpathways by considering experimentally validated miRNA‐target relationship, which were obtained from four common databases: miRTarBase,^18^ mir2Disease,^19^ miRecords^20^ and TarBase.^21^ The strategies of embedding miRNAs in subpathways have been utilized many times in our previous studies.^22^, ^23^ Moreover, the subpathways with less than 4 mRNAs and 3 miRNAs were removed. Finally, a total of 1602 subpathways, with an average of 20.8 mRNAs and 24.0 miRNAs, were taken into consideration for further analyses.

### 2.5 Calculating mRNA‐level and miRNA‐level subpathway activities

For mRNA‐level calculation, we computed the subpathway activity for each tumour sample based on high‐throughput mRNA expression using the FAMIE method.^15^ The major theory of FAIME is that a higher expression level of mRNA reflects a higher subpathway activity in which these mRNAs are involved. The detailed FAIME procedure to calculate mRNA‐level subpathway activity is showed as follows:

Step1: for each tumour sample, all expressed mRNAs were sorted in ascending order. A score based on the sorted order was assigned to each expressed mRNA. Step2: for each subpathway, we utilized the mRNA score from Step 1 to calculate a subpathway score, which was average mRNA score within this subpathway minus average score of all other mRNAs. Step3: considering the subpathway activity across all tumour samples, we further calculated the normalized activity of subpathways for all analysed samples. The detailed procedures and codes are derived from the study by Yang et al.[14]

In terms of miRNA‐level calculation, negative regulatory roles of miRNAs on target mRNAs were the most important things considered. The higher the expression levels of miRNA, the lower the subpathway activity in which the specific targeted mRNA was involved. Therefore, we revised the first step and ranked all expressed miRNAs in decreasing order. FAIME Step2 and Step3 procedures were the same as the mRNA‐level procedures. Finally, we also calculated the normalized subpathway activity at the level of miRNA for all analysed samples.

### 2.6 Survival analysis method

For single subpathway signatures, we performed Cox univariate analysis to calculate the prognostic significance and hazard ratio (HR) value. The significant signatures with HR > 1 denote risky factors, and the significant signatures with HR < 1 denote protective factors. For multiple subpathways, we clustered the tumour samples into two high‐risk and low‐risk groups based on risking score method. In detailed, for each sample from validation data set, we calculated risking score according to a linear combination of the subpathway activities weighted by the regression coefficients of corresponding subpathways. The coefficient and median risk score, as a threshold value derived from the training data set, were directly applied to validation data set to divide the samples into high‐risk and low‐risk groups. For the two risk groups, we further performed the Kaplan‐Meier (K‐M) analyses to compute the survival difference. Further, a log‐rank test was used to evaluate the prognostic significance of differences between groups. In all survival analyses, a *P*‐value < .05 was considered significant.

## 3 RESULTS

### 3.1 The overall analytical framework

In this study, we obtained sample‐matched mRNA and miRNA expression profiles of OvCa from TCGA database, as a training set, and ICGC database, as a validation set. The complete analysis was performed in three steps:

Step1: we utilized reconstructed subpathway and modified FAIME method to respectively calculate mRNA‐level and miRNA‐level subpathway activity for OvCa sample and formed corresponding subpathway activity matrix. Step2: randomly selecting half samples from training set for 1000 times, we utilized univariate Cox method to calculate prognostic significance and further obtained robust subpathway by counting the significant times in 1000. Meanwhile, we performed comprehensive analyses of OvCa prognostic subpathways between mRNA level and miRNA‐level. Step3: using the validation data set, we finally verified the predictive power of robust subpathway signatures and made a comparison among mRNA‐level results, miRNA‐level results and merged results. The overall framework is shown in Figure S1.

### 3.2 The analysis of method reliability

**Figure 1:**
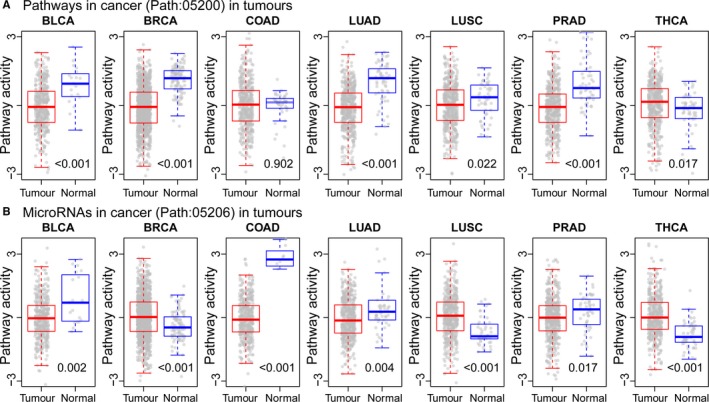
The tumour pathway activities between normal and tumour samples on seven tumour types. Based on the mRNA and miRNA expression profiles of seven TCGA tumour types, we utilized FAIME method to calculate the activity for Path: 05200 (A) and utilized the modified FAIME method to calculate the activity for Path: 05206 (B). For each tumour type, the difference of pathway activities between tumour and normal samples were compared and the significance value was calculated using Wilcoxon rank sum test

Before applying the modified FAIME method on OvCa prognosis, we performed an analysis to test the method reliability. Firstly, we obtained two specific pathways as golden cancer pathways: Path: 05200, which contained key cancer genes, and Path: 05206, which contained key cancer miRNAs. The difference of these two golden pathways between tumour and normal samples could reflect the effective performance of our method strategy. Owing to the absent of ovarian normal samples for comparison, we then downloaded and obtained sample‐matched mRNA and miRNA expression profiles from other seven tumour types (see Materials and Methods). Based on the high‐throughput expression profiles, we calculated the Path: 05200 activities for tumour and normal samples at the mRNA level using the FAIME method, and calculated the Path: 05206 activities at the miRNA level using the modified FAIME method (see Materials and Methods). For both Path: 05200 and Path: 05206, we observed the significant difference of pathway activities between tumour and normal samples in most tumour types (see Figure 1), which indicated the reliability of FAIME and modified FAIME methods for inferring mRNA‐level and miRNA‐level functional activity in following analysis.AUTHOR: Please suggest whether the term ‘Path’ can be changed to ‘path’ throughout the article.yes, the term "Path" can be changed to "path".

### 3.3 The comprehensive analyses of OvCa mRNA‐level and miRNA‐level prognostic subpathways

**Figure 2:**
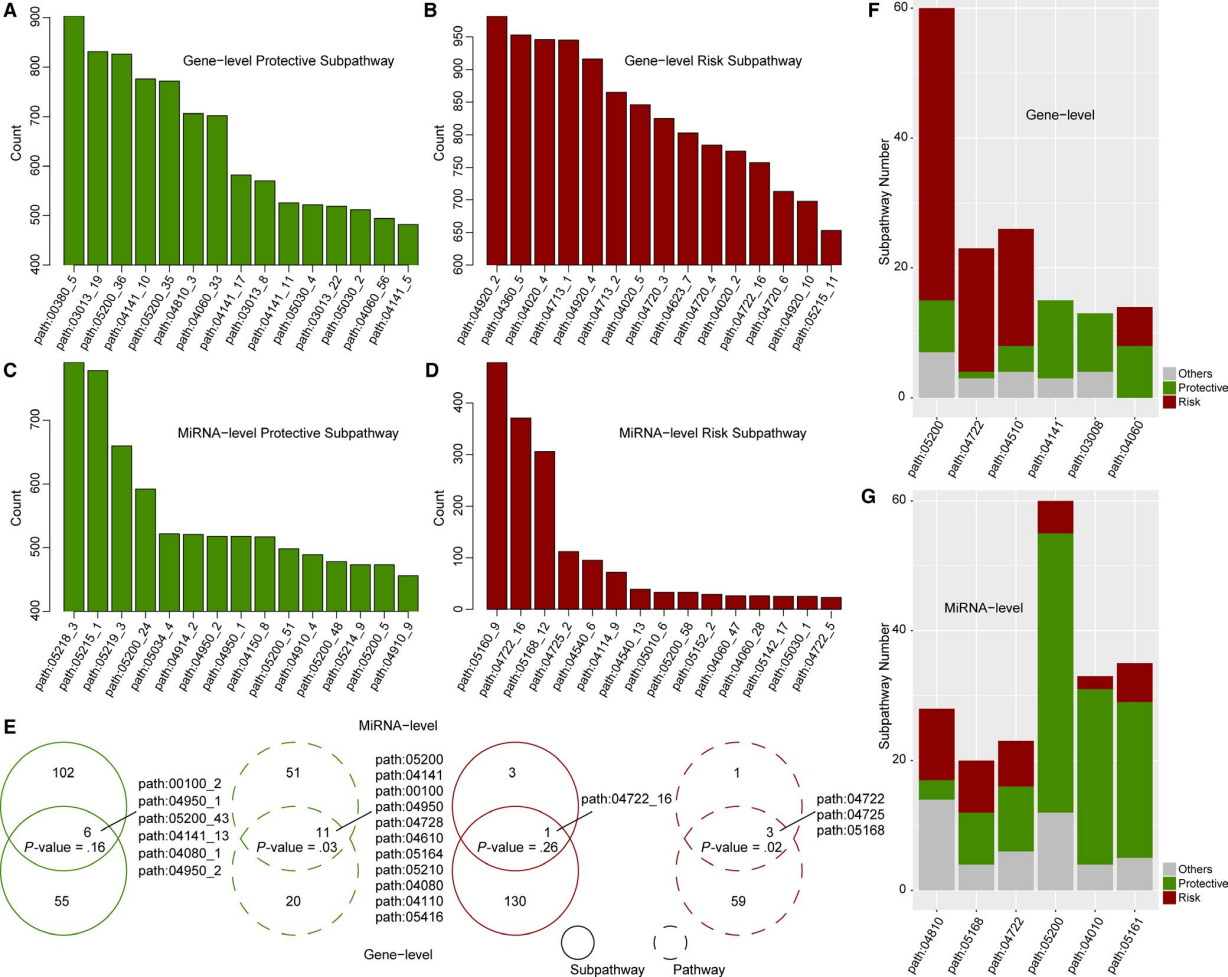
The comprehensive analyses of OvCa robust prognostic subpathways from mRNA level and miRNA level. The robust (top 15) protective subpathways (A) and risk subpathways (B) from mRNA level, and robust (top 15) protective subpathways (C) and risk subpathways (D) from miRNA level. (E) The subpathway and corresponding pathway comparison between mRNA level and miRNA level. The *P*‐values were calculated by hypergeometric test. Risk and protective subpathway distributions within some specific pathways from the mRNA level (F) and miRNA level (G)

Using the reconstructed subpathways and the mRNA/miRNA high‐throughput expression profiles of OvCa, we respectively formed mRNA‐level and miRNA‐level subpathway activity matrix. To obtain the most robust prognostic signatures, we performed 1000 sub‐training sets and assign each significant subpathway a number from 1 to 1000 (see Materials and Methods). Furthermore, two types of prognostic subpathways, risky and protective, were considered. The top 15 ranked risk and protective subpathways are shown in Figure 2A‐D. Overall, the mRNA‐level results displayed more robust trend than miRNA‐level results. For example, there were many more high robust subpathways (with counting number > 800) found on the mRNA level than the miRNA level. For mRNA‐level results, the risk subpathways displayed more robust trends. However, the miRNA‐level risk subpathways displayed opposite trends. There is only one subpathway's counting number greater than 400. So, the rank‐based and threshold‐based strategies should be both considered to define subpathway signatures for different distribution of mRNA and miRNA levels. The detailed results from two levels were provided as Tables [Link], [Link], [Link], [Link].

For the prognostic subpathways with the same risk directions, we further performed a systematic comparison between mRNA level and miRNA level. As shown in Figure 2E, for protective subpathways with numbers greater than 200, six overlapping subpathways were shared by 61 subpathways from the mRNA level and 108 subpathways from the miRNA level with hypergeometric *P*‐value = .16. Similar results were observed for risk subpathways. Only 1 subpathway was shared by 131 subpathways from mRNA level and four subpathways from miRNA level with hypergeometric *P*‐value = .26. Furthermore, we transferred the subpathway results into pathway results, and the different subpathways derived from the same whole pathway were regarded as the same pathway results. At the pathway level, the 31 mRNA‐level pathways and 62 miRNA‐level pathways shared significant 11 overlapping pathways (hypergeometric *P*‐value = .03). Some pathways including path: 04782, path: 04610, path: 05164, path: 05210, path: 04110 and path: 05416 were recognized from pathway level, but not from subpathway level. For risk pathways, we also observed the consistent results: three pathways including two new pathways (path: 04725 and path: 05168) were significantly shared with hypergeometric *P*‐value = .02. Therefore, we conclude that the mRNA‐level and miRNA‐level prognostic subpathways tend to be located in the different regions of the same pathways, which reflecting the function‐level mutual exclusion from two different levels. From a biological point of view, it is unlikely to destroy the detailed subpathway region twice, from mRNA level and miRNA level. For detailed comparison of each prognostic subpathway result between mRNA level and miRNA level, we also observed the mRNA‐ and miRNA‐level mutual exclusion for prognostic subpathways (see Figure S2).

For some specific pathways, we respectively made statistics on the proportion of risk subpathways, protective subpathways and others (see Figure 2F‐G). For example, most subpathways of Path: 05200 (Pathways in cancer) were risk subpathways at mRNA level whereas most subpathways of this pathway were protective ones at miRNA level. Again, we observed the opposite trends between mRNA‐level and miRNA‐level subpathway results. At the mRNA level, there were no risk subpathways between path: 04104 and path: 03008. All the subpathways in path: 04060 were related with the OvCa prognosis. Moreover, there are more protective subpathways than risk ones. In this part, it is also confirmed that the subpathway scale displayed more sensitive than pathway scale.

### 3.4 The first verification: rank‐based subpathway signatures

**Figure 3:**
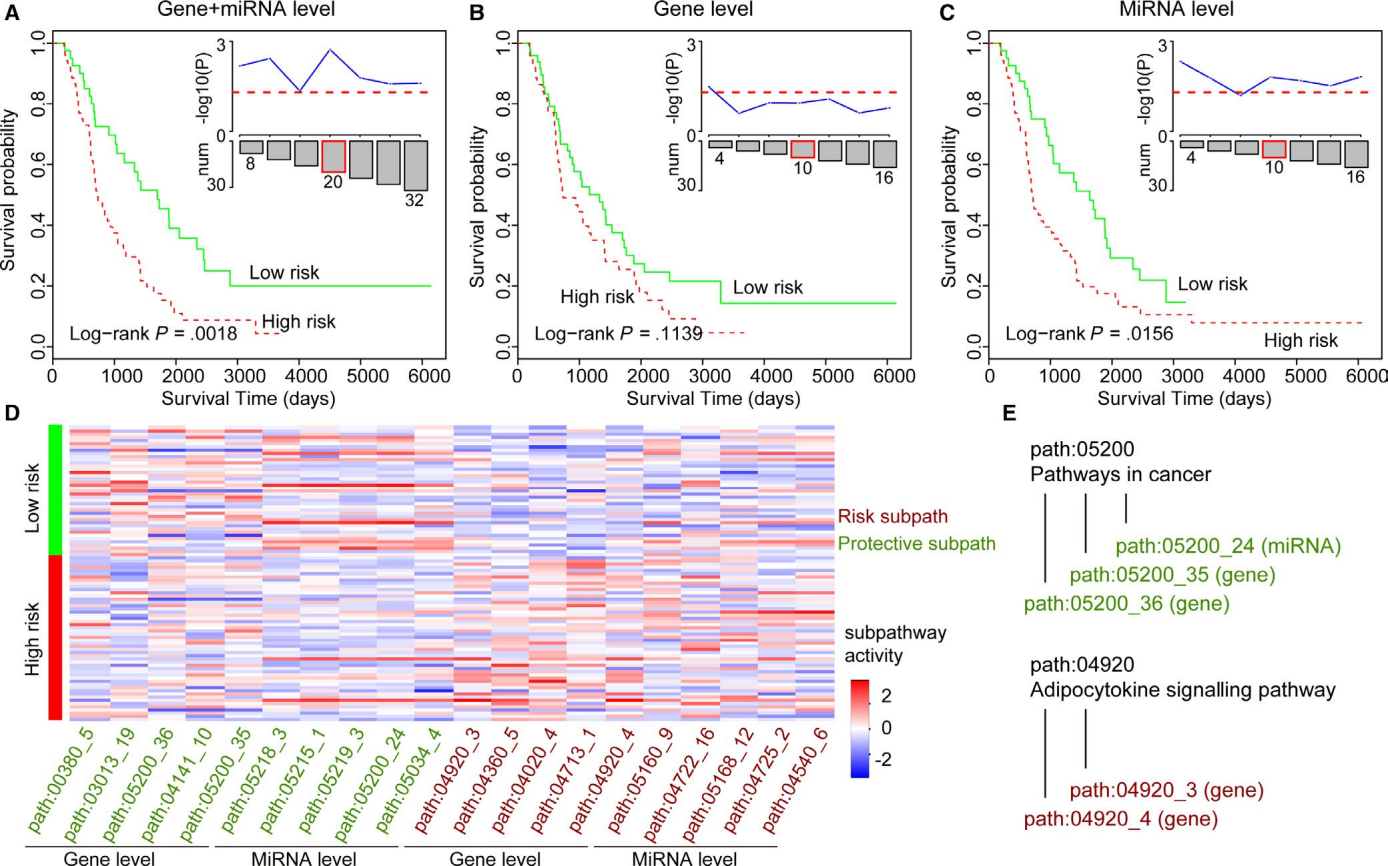
The rank‐based verification in the validation set. The K‐M curves of the combined 20‐subpathway signatures with top 5 risk and protective subpathways from mRNA and miRNA levels in (A). The corresponding mRNA‐level results in (B). The corresponding miRNA‐level results in (C). The different top rank results from top 2 to 8 were shown in top‐right of Figure 3A‐C. (D) The heatmap of top 5 risk and protective subpathways from each level. Red indicates the high subpathway activity, and green indicates the low subpathway activity. (E) Take two specific pathways as examples to show prognostic subpathway relationships within the same pathway

The OvCa prognostic subpathways shared few overlaps between mRNA‐level and miRNA‐level results, as previously stated. Thus, we tested the predictive performance of the combined signatures from top‐ranked strategy using an independent validation set from ICGC database (see Materials and Methods). Utilizing the same method as training set, we also formed mRNA‐level subpathway matrix and miRNA‐level subpathway matrix for ICGC data set. Next, top‐ranked risk and protective subpathways from both mRNA‐level and miRNA‐level results were considered as combined signatures, and risking score method was applied based on these subpathways to form two different sample clusters, low‐risk and high‐risk. Finally, a log‐rank test was used to evaluate the survival difference between two sample clusters and *P*‐value significance was calculated. Take the top 5 as an example; we obtained a total of 20 subpathways as the combined prognostic signatures with 5 from risk mRNA‐level results, 5 from protective mRNA‐level results, 5 from risk miRNA‐level results and 5 from protective miRNA‐level results. As shown in Figure 3A,D, the top 20‐subpathway combined signature displayed significant predictive performance in the validation set with *P*‐value = .0018. Notably, the mRNA‐level and miRNA‐level risk subpathway displayed higher activities in the high‐risk samples than low‐risk samples, especially the mRNA‐level results. Comparatively, protective subpathways from two levels displayed higher activities in the low‐risk samples than high‐risk samples. All these evidences were displayed in the ICGC validation data set and showed the consistent prognostic trends with results from training set. Taking the Pathway in cancer (Path: 05200) as an example, two mRNA‐level subpathways (Path: 05200_35 and _36) were identified as protective factors, as well as another miRNA‐level subpathway (Path: 05200_24) was also identified (see Figure 3E). And it was also consistent with previous conclusions that mutual exclusion of mRNA‐level and miRNA‐level results.

To test whether the combined signatures displayed advantages over individual signatures from only mRNA‐level or miRNA‐level, we performed a systematical comparison. For the subpathway signatures identified only from mRNA‐level or miRNA‐level, we also utilized the same methods to evaluate the predictive performance of corresponding subpathway signatures. Take also top 5 as an example, the 10 subpathways from mRNA level and 10 subpathways from miRNA level were obtained and compared with combined results. As shown in Figure 3B,C, the observed mRNA‐level signatures (n = 10) were not related with the OvCa prognosis (*P*‐value = .1139), while the miRNA‐level signatures (n = 10) were related with OvCa prognosis (*P*‐value = .0156), which did not reach the significance value of combined 20‐subpathway signatures. To show the robustness of prognostic performance, we further performed a series of analyses by defining different top number from 2 to 8. At the different threshold of top number, we respectively obtained combined signatures and individual ones and performed the survival analysis. As shown in top‐right inner figures of Figure 3A‐C, the combined signatures displayed more prognostic significance results than individual ones, further confirming the necessary of integrating mRNA and miRNA levels.

### 3.5 The second verification: threshold‐based subpathway signatures

**Figure 4:**
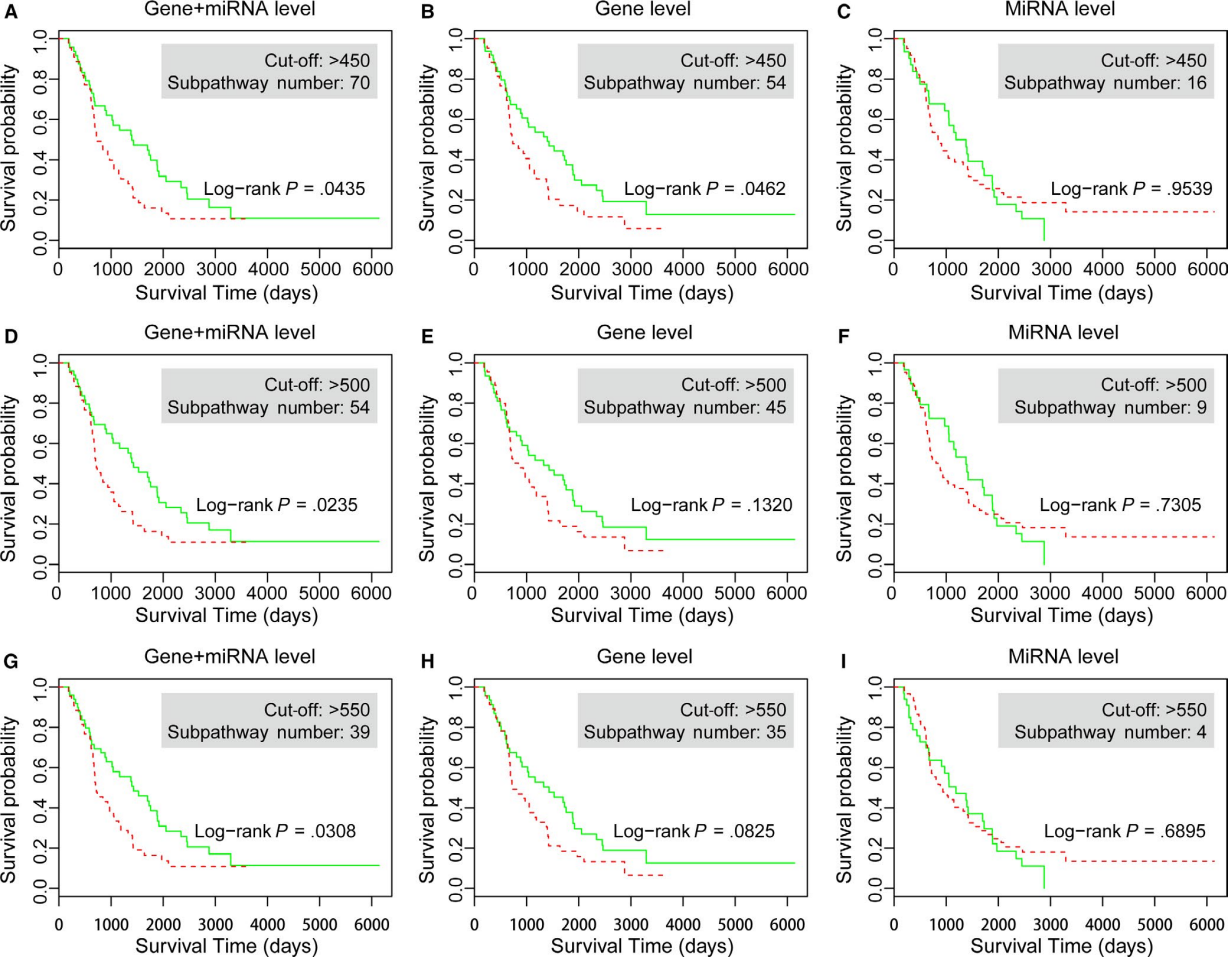
The threshold‐based verification in the validation set. The K‐M curves of the combined signatures with three cut‐offs (450, 500 and 550) from each mRNA or miRNA level. (A) combined 70 signatures, (B) mRNA‐level 54 signatures, (C) miRNA‐level 16 signatures in threshold 450, (D) combined 54 signatures, (E) mRNA‐level 45 signatures, (F) miRNA‐level 9 signatures in threshold 500, (G) combined 39 signatures, (H) mRNA‐level 35 signatures and (I) miRNA‐level 4 signatures in threshold 550

The mRNA‐level and miRNA‐level results displayed different distribution in 1000 sub‐training analysis (see Figure 2). Therefore, we performed another verification, threshold‐based strategy, to define prognostic subpathways and test the predictive performance. Considering the suitable number of signatures for prognostic research, we totally selected three thresholds, 450, 500 and 550, in this verification. The detailed number of subpathway signatures of mRNA‐level and miRNA‐level based on these three cut‐offs are shown in Figure S3. Utilizing the same methods as mentioned above, we obtained the prognostic subpathways and calculated the predictive significance of these signatures. Also, the comparison between combined signatures and individual signatures was performed. As shown in Figure 4, the combined subpathway signatures displayed significant predictive power at three cut‐offs (*P*‐value = .0435 in threshold 450, *P*‐value = .0235 in threshold 500 and *P*‐value = .0308 in threshold 550). However, the mRNA‐level signatures displayed significant predictive power only in one condition (*P*‐value = .0462 in threshold 450), and miRNA‐level signatures displayed no significant predictive power in all thresholds. All these evidences further confirmed the stronger predictive performance of combined mRNA‐miRNA signatures than individual ones and the effective ability to identify prognostic signatures by our novel integrated strategy.

To test the predictive robustness of combined signatures, we further performed a comparison with random signatures. In detailed, we randomly obtained signatures from risk and protective subpathway results from both gene‐level and miRNA‐level, and the random subpathway number was the same as combined signatures. The random process was performed in 1000 times. Take the top 5 signatures as an example, we respectively obtained 5 random subpathways from all gene‐level risk results, 5 from gene‐level protective results, 5 from miRNA‐level risk results and 5 from miRNA‐level protective results. Then, a total of 20 random subpathways were obtained and the predictive significance was calculated using the validation set for each random in 1000 times. In the meanwhile, the random results based on threshold strategy were also performed. As shown in Figure S4, the combined signatures displayed more predictive power than random signatures in both rank‐based and threshold‐based strategies.

## 4 DISCUSSION

In this research, we respectively inferred subpathway activity from mRNA and miRNA expression levels and performed a bootstrap analysis to identify the most robust subpathway signatures for OvCa prognosis. Because of the different roles of mRNA and miRNA molecules, we utilized different methods to infer the functional activity, and confirmed the effectiveness of these methods. For OvCa application, we observed the mutually exclusion evidence of subpathway dysregulation between mRNA and miRNA levels, which indicated the necessary of combination of results from multiple levels. Finally, we validated the predictive performance of combined subpathway signatures using two strategies using the independent ICGC data sets. The survival comparison was performed between combined signatures and individual signatures only from mRNA level and miRNA level.

Based on high‐throughput mRNA and miRNA expression profiles, the subpathway activity was calculated for each tumour sample using the previous FAIME method.^15^ This method was developed to infer activity for functional sets including biological pathways or GO terms. The genes within functional set were considered as a whole, and the overall expression condition compared to genome‐wide expression level was calculated. It was confirmed that FAIME method analysis was more effective than other methods. Recently, the subpathway concept was derived and defined in the study of Li et al.^17^ Compared to the whole pathway, subpathways were located in detailed regions involved in the pathway and contained smaller sets of genes. The pathway usually contained many subpathways, and the different subpathways within the same pathway often exhibited totally different biological meanings.^17^, ^24^ Genes within the same subpathway usually displayed the more consistent expression pattern than pathway level. Moreover, the subpathway concept was applied into many aspects of researches, including miRNA regulation and disease,^25^ and tumour prognosis analysis.^22^, ^26^ Concurrently, in this study, FAIME method took less genes within subpathway set to infer more detailed activity value for subpathway. Therefore, the application of FAIME method on biological subpathway should be the more proper strategy to infer functional activity for clinical use.

For identifying the most robust prognostic signatures, we performed the bootstrap to form 1000 sub‐training sets based on the TCGA training set. For each sub‐training set, we performed univariate Cox analysis to identify prognostic subpathways with *P*‐value < .05. A counting number from 1 to 1000 was assigned to each prognostic subpathway by integrating all 1000 bootstrap results. In detail, we further considered the impact of prognostic signatures and defined risk or protective signatures according to the HR value. Therefore, a count value was assigned to each risk and protective subpathways for results from both mRNA and miRNA levels, and all these subpathways were also ranked in a descending order. Owing to the difference of mRNA‐ and miRNA‐level result distribution, we performed two different strategies (rank‐based and threshold‐based) to obtain the final prognostic subpathways. For the rank‐based strategy, the 20‐subpathway signatures were obtained from mRNA and miRNA levels at the top 5 rank. For the threshold‐based strategy, the 70‐subpathway signature was obtained at the threshold 450. Considering all these strategies, we confirmed the performance of mRNA‐miRNA combined signatures and the advantage over results from individual mRNA or miRNA levels.

For both mRNA and miRNA levels, these top‐ranked subpathways contained some tumour‐related pathways (pathways in cancer, RNA transport) and specific pathways, most of which displayed close relationship with OvCa prognosis and biological mechanism. For path: 00380_5 (Tryptophan, top 1 in mRNA protective results), it was observed that patients with OvCa have increased tryptophan degradation compared to controls resulting in higher serum kynurenine concentrations,^27^ and elevated Kyn/Trp levels were shown to be associated with OvCa poor response to therapy and worse outcome.^28^ For path: 04810_3 (Regulation of actin cytoskeleton, top 6), study had reported the dysregulation of the cytoskeleton during OvCa progression in a mouse model.^29^ Moreover, the abnormal expression of Beta‐actin (ACTB) and the resulting changes to the cytoskeleton were associated with tumour invasiveness and metastasis.^30^ In another study, the cytokine‐cytokine receptor interaction, in which the path: 04060_33 (top 7) was involved, was also enriched in the OvCa long survival event.^31^ For mRNA‐level risk results, the overexpression of retinol binding protein 4 (RBP4) in OvCa cells promoted cancer cell migration and proliferation,^32^ and an adipokine secreted by adipose tissue also induced cell invasion and metastasis,^33^ which showed the potential risk roles of path: 04920_2 (Adipocykine signalling pathway, top 1). For path: 04360_5 (Axon guidance, top 2), a previous transcriptome‐based study also revealed the axon guidance molecules were related with OvCa clinical prognosis.^34^ Furthermore, CaMKK2 (one key kinase in calcium signalling) knockdown potentiated the effects of the chemotherapeutic drugs carboplatin and PX‐866 to reduce OvCa survival.^35^ Although miRNA‐level subpathway results displayed lower rank than mRNA level, most top‐ranked subpathways were also associated OvCa clinical mechanism. Among the miRNA‐level protective results, we identified path: 04950_2 (Maturity onset of diabetes of the young, top 7). Recently, it has been shown that the expression of hepatocyte nuclear factor‐1beta (HNF1β) was associated with OvCa risk.^36^ Another important pathway, mTOR signalling pathway (top 9) was considered as a promising therapeutic target in OvCa treatment in the recent study.^37^ Among the miRNA‐level risk results, Hepatitis C (top 1) and Herpes simplex infection (top 3) were both identified, and many evidences revealed the association between infectious diseases and OvCa or other tumours.^38^, ^39^, ^40^ In addition, two integrative analyses both showed that oocyte meiosis (path: 04114_9, top 6) was significantly enriched in the OvCa differentially expressed genes.^41^, ^42^

We developed a novel mRNA‐miRNA integrated framework to identify robust prognostic signatures for OvCa based on the TCGA data set, and further verified the survival predictive performance of combined signatures using independent ICGC data set. The survival verification was performed based on the high‐throughput sample‐matched mRNA and miRNA expression profiles, which are limited and recently increasing. With more available data sets, the framework of our integrated framework will gain more confirmation. In future research, we will further consider multiple levels of information to optimize core mRNA or miRNA molecules within key subpathway signatures.

## CONFLICT OF INTEREST

The authors declare that there are no conflicts of interest.

## AUTHOR CONTRIBUTIONS

ST, WM and LX conceived the study, acquired the data, and interpreted the data. ST, MZ and CZ wrote the manuscript and revised it critically for the important intellectual content. All authors approved the final version to be published.

## Data Availability

All data utilized in this study are included in this article.
