# Supplementary figures and images for: Comprehensive analysis of mRNA‐level and miRNA‐level subpathway activities for identifying robust ovarian cancer prognostic signatures
Source: J Cell Mol Med. 2020 Jan 19;24(4):2582–92. doi: 10.1111/jcmm.14968 (PMC7028850; doi:10.1111/jcmm.14968)

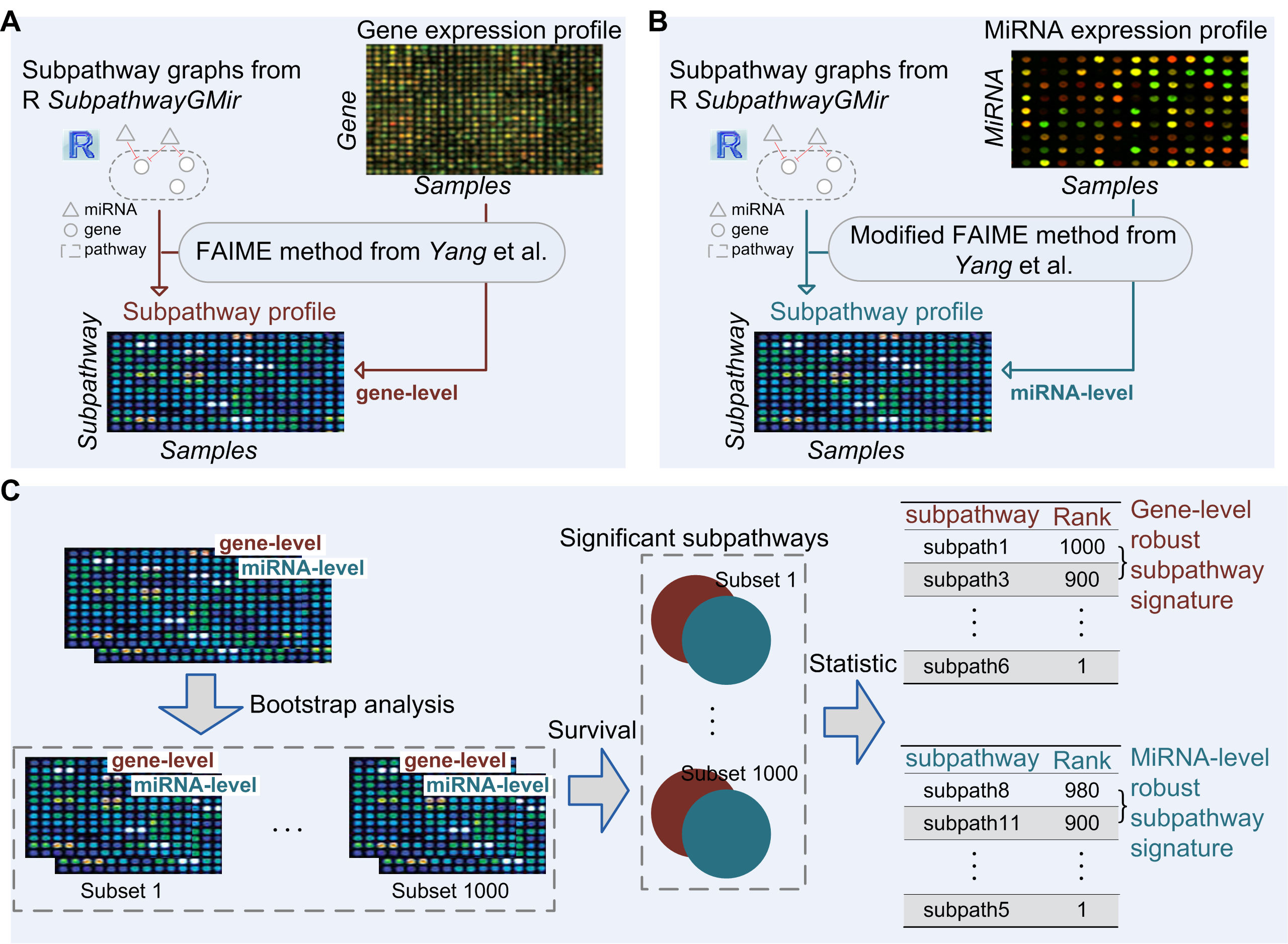

Supplement: Supplementary file 1 [file JCMM-24-2582-s001.tif]

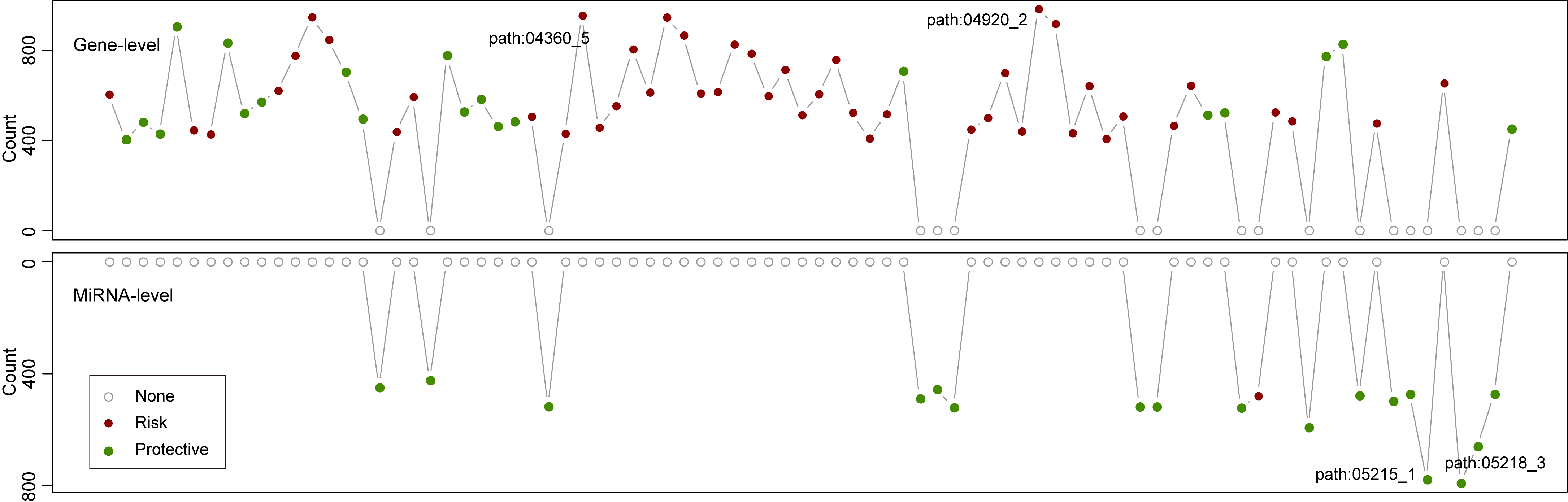

Supplement: Supplementary file 2 [file JCMM-24-2582-s002.tif]

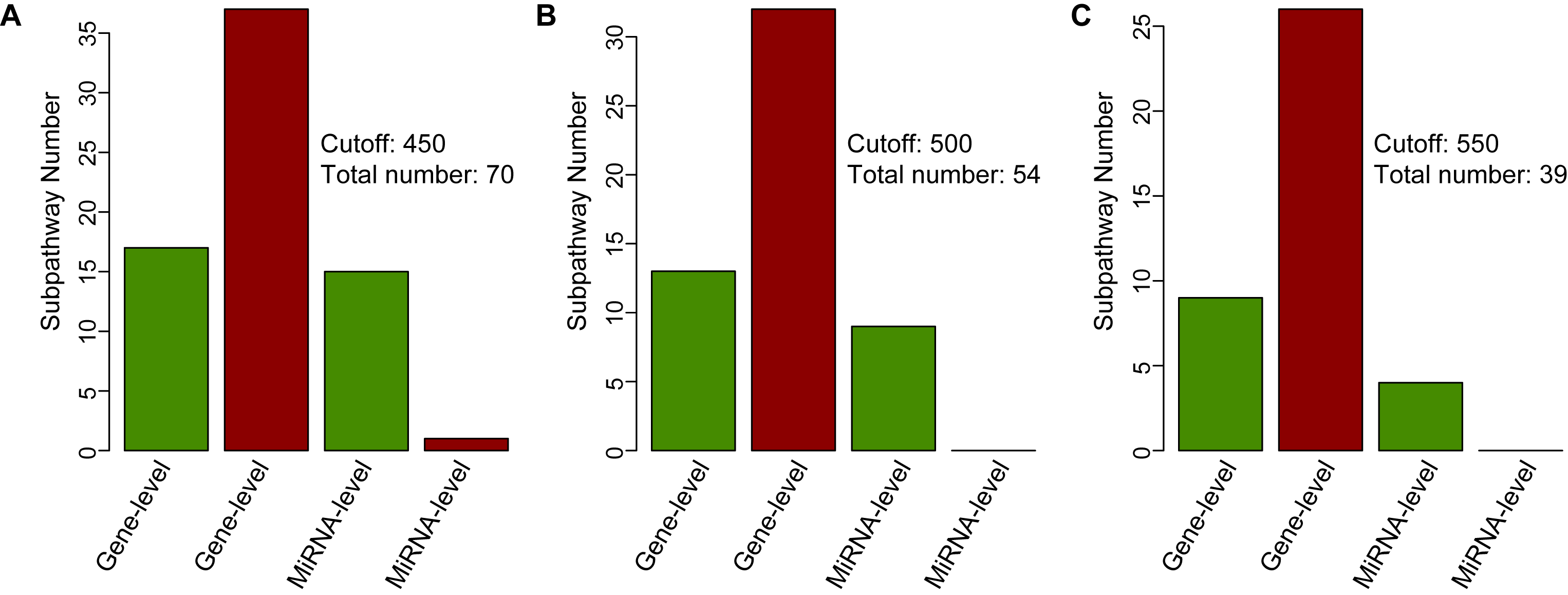

Supplement: Supplementary file 3 [file JCMM-24-2582-s003.tif]

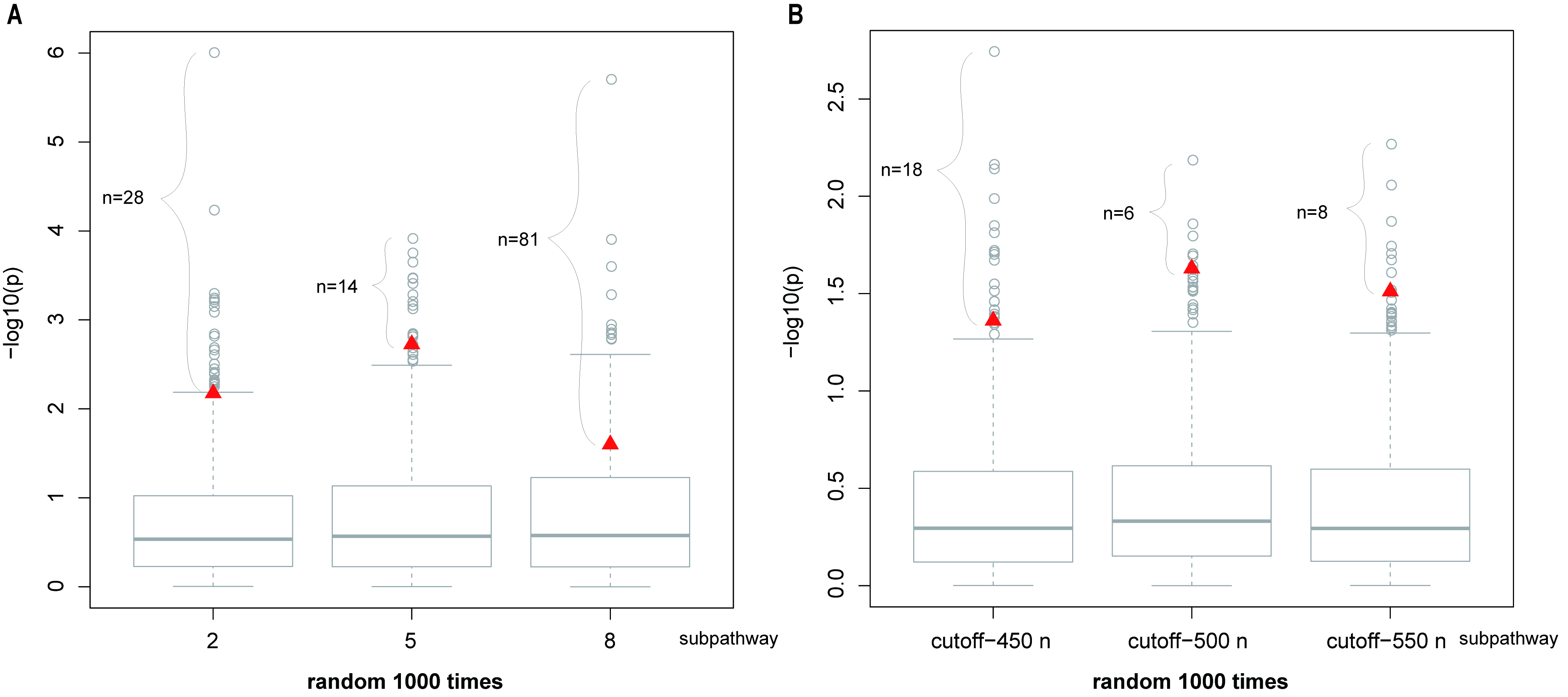

Supplement: Supplementary file 4 [file JCMM-24-2582-s004.tif]
